# Application of Molecular Dynamics Simulations in the Analysis of Cyclodextrin Complexes

**DOI:** 10.3390/ijms22179422

**Published:** 2021-08-30

**Authors:** Anna Helena Mazurek, Łukasz Szeleszczuk, Tomasz Gubica

**Affiliations:** 1 Department of Physical Chemistry, Chair of Physical Pharmacy and Bioanalysis, Faculty of Pharmacy, Doctoral School, Medical University of Warsaw, Banacha 1 Street, 02-093 Warsaw, Poland; anna.mazurek@wum.edu.pl; 2 Department of Physical Chemistry, Chair of Physical Pharmacy and Bioanalysis, Faculty of Pharmacy, Medical University of Warsaw, Banacha 1 Street, 02-093 Warsaw, Poland; tomasz.gubica@wum.edu.pl

**Keywords:** cyclodextrins, molecular dynamics, MD, host–guest complexes, simulations

## Abstract

Cyclodextrins (CDs) are highly respected for their ability to form inclusion complexes via host–guest noncovalent interactions and, thus, ensofance other molecular properties. Various molecular modeling methods have found their applications in the analysis of those complexes. However, as showed in this review, molecular dynamics (MD) simulations could provide the information unobtainable by any other means. It is therefore not surprising that published works on MD simulations used in this field have rapidly increased since the early 2010s. This review provides an overview of the successful applications of MD simulations in the studies on CD complexes. Information that is crucial for MD simulations, such as application of force fields, the length of the simulation, or solvent treatment method, are thoroughly discussed. Therefore, this work can serve as a guide to properly set up such calculations and analyze their results.

## 1. Introduction

Since the 1970s, there has been a rapid increase of interest in the industrial application of cyclodextrins (CDs) [1]. This growth was associated with an unambiguous confirmation of the nontoxicity and considerable price decrease of CDs. CDs were earlier considered as “toxic”, as mistakenly ascribed by French in 1957 [2]. Fortunately, Szejtli postulated the lack of toxicity, a view that was thoroughly examined and, finally, widely accepted [3,4]. Since then, the price of CDs has rapidly dropped; they currently cost as low as USD 5 per kilogram [5].

CDs are primarily used in pharmaceutical formulations due to their unique properties [6]. Through the formation of host–guest complexes, they increase the solubility of poorly soluble drugs and protect substances against external factors, such as light, humidity, and heat. Moreover, CDs could mask unpleasant smells or flavors of drugs, which is especially important in formulations dedicated to children. Currently, more than 100 original drugs are manufactured with CDs as excipients [7,8,9]. Interestingly, CDs play a role in fighting the COVID-19 pandemic. For example, CDs can be found in formulations of remdesivir [10] (an antiviral drug administered to treat COVID-19) and Johnson & Johnson’s Jannsen (J&J/Janssen) single-shot COVID-19 vaccine [11]. CDs are also utilized in the production of face masks, which are widely used to help slow the spread of the coronavirus [12].

The desirable properties of CDs in the pharmaceutical field can be explained at the molecular level [13]. Cyclodextrin (CD) molecules resemble a “doughnut” ring, in which small, non-polar substances can be entrapped. The external fragments of CD molecules are polar due to the presence of hydroxyl groups. When a non-polar substance (e.g., a poorly soluble drug) enters the molecular hole of cyclodextrin, the formed host–guest complex is polar (at outside) and, therefore, is more soluble than the separated guest molecule. 

Solid complexes of active pharmaceutical ingredients (APIs) with CDs are amorphous, in major cases [14]. Therefore, one can conclude that they are characterized by high disorder and dynamics. Hence, molecular dynamics (MD) simulations seem to be an ideal tool to investigate their structure and properties.

The aim of this work was to review the articles presenting results of MD simulations of the host–guest CD complexes and provide an accessible introduction to those calculations for non-experts. While MD simulations can provide information unobtainable by any other methods, they can also be very demanding. For years, the high computational costs of these simulations prevented their application in this topic, as well the need for proper knowledge and experience to set up such calculations and correctly analyze the obtained results. However, we believe that our overview of successful MD simulation applications could facilitate this task and inspire the reader to take advantage of the unique opportunities offered by them.

## 2. Molecular Modeling of CD Host–Guest Complexes—Theoretical and Practical Aspects

As above-mentioned, this review aims to present the results of MD simulations on CD host–guest complexes to study their structures, properties, and inclusion processes. However, apart from MD, other techniques have been widely utilized in modeling CDs, i.e., molecular docking, quantitative structure–activity/property relationships (QSARs/QSPRs), Monte Carlo simulations, and machine learning methods. Those approaches have been briefly described in general reviews, focusing on application of molecular modeling methods in this field [15,16]. Therefore, they will not be discussed in this article. Moreover, the benefits resulting from encapsulation of various molecules in the form of CD complexes will not be described here, as there are other (recent and excellent) reviews on this topic [17,18,19]. Therefore, this paper will begin with a brief summary of the concepts of MD simulations, followed by solutions that are frequently used to properly model the CD complexes by its means. The main part of this work will be the presentation of the published results and discussions of the most interesting, state-of-the-art examples, in order to draw general conclusions.

### 2.1. MD Simulations—A Perfect Choice to Study CD Complexes

A MD simulation is a well-established technique used for the study of various molecules, complexes, and mixtures, in any state of matter, and at almost any temperature and pressure condition [20,21]. MD methods are sometimes described as “computational molecular microscope(s)” as they provide means that enable understanding the structure and behavior on a molecular level. In fact, MD is more than just an in silico microscope as it can be used to determine structural, energetic, and thermodynamic properties [22]. Further, the snapshots obtained from the MD simulations can be used, i.e., to increase the accuracy of the calculations of spectroscopic parameters [23].

While designing and preparing the CD complexes, the formation of the covalent bond between host and guest molecules is usually avoided in order to enable the guest to freely escape from the CD cavity. Therefore, CD host–guest complexes are stabilized by non-covalent interactions, such as H-bonds and van der Waals forces (vdW). In most cases of such complexes, a few energetically-similar minima, found as the creation of one bond, usually requires the breaking of another of similar strength. This is also in agreement with the experimental results—the solid complexes of CDs are usually amorphous, as the guest molecule can exist in various conformations and in various poses. Therefore, modeling static structures of CD complexes through geometry optimization, without considering their dynamics, can be a source of inaccuracy, and significant differences between the experimentally and computationally obtained results. Hence, as will be proven in the next section, MD simulation is a perfect method to study CD complexes.

MD methods, regardless of the objects modeled and the method used, are, in their basic assumptions, similar. The simulation starts with an initial configuration of the system and energy minimization through the optimization of the positions of all atoms. This is usually a prerequisite for MD simulations, otherwise the excess potential energy would transform to kinetic energy and the simulation would be unstable or longer equilibration would be necessary [24]. Subsequently, the forces acting on each atom are calculated and used in equations of motion to update the configuration. This process is repeated to generate a trajectory—a contiguous set of configurations obtained during the time evolution of a studied system. Moreover, MD simulations can be enhanced by adding external potentials, which will be described in Section 2.5.

For MD simulations of large molecular complexes, such as ligand–protein, molecular mechanics (MM) methods are commonly used. On the contrary, when MD simulations are performed on relatively small molecules, it is usually at the quantum mechanics (QM) level of theory, which significantly increase the accuracy of calculations, but also their computational costs. In terms of the sizes of the modeled objects, CD complexes are somewhere in between. While geometry optimization calculations on the static structures of CD complexes are, nowadays, performed mostly at the QM level, usually by the means of DFT [25], the MD simulations are still being performed at the MM level. However, to increase the accuracy of the calculations, while still maintaining their reasonable computational costs, multiple solutions, such as dedicated force fields and special sampling methods, have been developed, and will be described below.

### 2.2. Force Fields Dedicated to Cyclodextrins

The literature review provides an overview on the force fields (FFs) used in the MD simulations of CD complexes. Often, universal FFs, such as Generalized Amber FF (GAFF) in AMBER [26], GROMOS96 in GROMACS [27], CHARMM in CHARMM [28] and NAMD [29], OPLS2005 in Desmond [30], or COMPASS in Forcite [31], are used, the first three being the most popular in this topic. The data on applied software and FFs are presented in Table 1.

However, to increase the accuracy of the simulations, the dedicated FFs have been parametrized and validated. This was possible due to the structural similarities between the CDs, as all of them are composed of glucose units and the substituted ones possess similar functional groups. In the cases where these dedicated FFs are applied, the guest is treated with a standard FF (usually it is GAFF) and the CD with either Glycam06 FF [38] (for native CDs) or q4md-CD [39] (for substituted CDs). The former does not contain parameters that describe delocalized atoms so these data are taken from GAFF. The latter is a combination of Glycam04 and Amber99SB; thus, still being based on the CD-specific FF, it takes into account additional substitutions at the hydroxyl groups of CDs. Combining this information—common FFs applications for the MD calculations of CD complexes would either be GAFF + Glycam06 or GAFF + q4md-CD. 

While many MD simulations of CD complexes are performed using a single FF, sometimes it is necessary, or preferable, to combine them. However, such an approach requires the compatibility of those FFs in a validation form that should be performed on the simulation. Since it is likely that any given researcher is using MD to study some previously unstudied molecule, then it is not possible to predict, with absolute certainty, how the simulation will perform. Therefore, any combination of FFs and simulation conditions should be validated. For example, the GLYCAM force fields are designed to be independent of other biomolecular force fields, and, therefore, to work (at least reasonably) well with any of them, while CHARMM FFs are better suitable for combining with the CHARMM General Force Field [40].

Sometimes a different strategy is implied, namely Glycam06 for the CD molecule, whereas the partial atomic charges of a guest are derived on the basis of an approach, repetitively called a ‘standard parametrization procedure’ (see in Table 2: I.D. (CDs used as drug carriers (water environment)—plant-derived substances) N° 9, 10, 11, and 14 and I.F. (CDs used as drug carriers (water environment)—umbrella sampling and steered (biased) molecular dynamics) N° 6).

**Figure 1:**
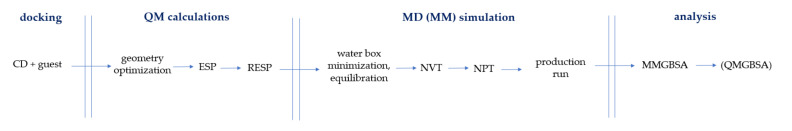
Preparation steps, performing and analysis of MD simulations of CD complexes (based on the gathered CD–MD literature).

This standard procedure is composed of several steps, visualized as a chart in Figure 1. First, the geometry of a structure is optimized on the quantum mechanical (QM) level of theory. In the majority of cases, DFT calculations are applied here. Then, the electrostatic potential (ESP) around the molecule is calculated [41]. However, fitting of the classical Coulomb model for the electrostatic potential into the QM ESP calculations results in ESP charges, which overestimate the strength of hydrogen bonds [42]. Therefore, a fitting (restraining) procedure must be performed, and based on the ESP charges, the restrained ESP (RESP) charges are obtained [42]. Later, the missing bonded parameters are derived from calculations with GAFF. 

A completely different approach is MM3 FF, which, in 1989, was called ‘a force field for hydrocarbons’ [43]. Later, in 2010, it was compared with Glycam06 FF and defined as far less accurate [44]; the newest computational approaches and the increasing computational power shed new light on this abandoned carbohydrate FF. In 2020, an article applying the MM3 FF for the MD simulation of CD and sertraline was published [45]. According to our best knowledge, it is the only example of MM3 application in the CD MD simulations.

There are two FF strands [46]. The first involve those constructed with the aim of modeling big systems, such as proteins. This group includes AMBER, CHARMM, and OPLS FF families. The second strand is composed of MM1, MM2, MM3, and MM4 FFs, which have been parametrized against experimental data and developed to provide accurate predictions of molecular structures and properties [46]. As a result, the application of such FFs is much more computationally expensive. As stated above, among all gathered from the articles in this literature review, there is only one in which MM3 FF is used [45]. These calculations are run with the Tinker code [47]. It is worth noting that the article was published very recently, in 2020, and that the Tinker-HP is a massively parallel MD package that is being strongly developed to calculate computationally expensive runs, such as long polarizable MD or polarizable self-consistent QM–MD simulations [48]. Therefore, one can expect further developments in computational possibilities. It is especially interesting from the CD calculation point of view, due to its relatively small size when compared with ligand–protein complexes or biological membranes. Therefore, in the nearest future, CD complexes are likely to be calculated using the QM–MD approach even more frequently.

### 2.3. MD Simulations of CD Complexes in Water Environment

In the reviewed studies, a pre-requisite for each MD simulation involved the creation of a complex between the CD and a guest molecule. To the authors’ best knowledge, so far, there have been no published attempts to simulate this process other than through the docking procedure. In other words, we found no work in which the process of the formation of the complex from substrates, CD, and guest molecules, would be studied using MD simulations. This was probably due to the long simulation needed to observe this reaction. The literature review shows that the most often used docking software are open-source AutoDock Vina [49] and commercial CDOCKER from the Accelrys Discovery Studio Package [50]. Surprisingly, the authors of some of the reviewed works underestimate the docking step and do not describe it with sufficient details. The proper initial orientation of the guest inside the CD cavity is particularly important to the linear molecules that may form two substantially different complexes: parallel and antiparallel. During classical MD simulations, it is almost impossible to observe the transition between those two complexes, as it would require complex dissociation, guest reorientation, and subsequent association of the guest and CD.

**Figure 2:**
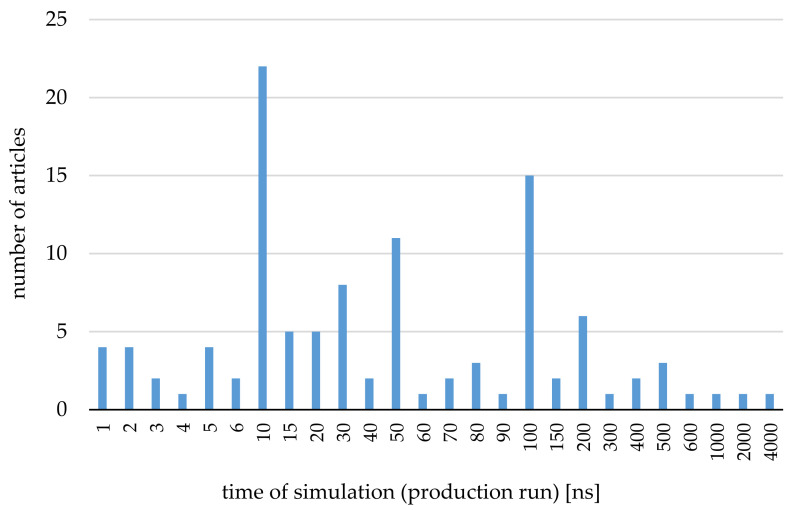
Time of production run in MD simulations of CD complexes used in articles in the period of 2012–2021.

Several steps for each MD simulation should be followed (Figure 1). First, a water box is built and water molecules undergo relaxation. This means that a constraint is put on the motion of heavy atoms (other than hydrogens). Then, the minimization of the system occurs and afterward an equilibration takes place. It starts under an NVT ensemble, which results in both stabilization of a box density as well as a “heating up” to the temperature of the production run. If the production run is to be performed under constant pressure, then the equilibration under the NPT ensemble is performed. A system prepared in this way can undergo a production run. The length of a production run depends on the time in which an equilibrium is reached, which in turn is a derivative of the system’s fluctuations degree. As the length of the MD simulation is entirely dependent on the system used, it is not possible to precisely determine, during the setup, how many nanoseconds (at the least) it would last. This can be checked during or after the production run, i.e., by the RMSD analysis. However, one can suspect that the similar objects would require a similar simulation time. As this review should assist those wanting to perform MD simulations on their own CD complexes, the information on simulation time is presented in Table 2. Additionally, an illustrative chart was prepared (Figure 2) for better visualization. According to this diagram, the time of the production run varies between 1 and 4000 ns, but in most of cases, it takes a value of 10 or 100 ns. 

In all molecular modeling studies, several approximations and constraints are imposed to properly simulate the real reaction environment and maintain the high accuracy of the results in a reasonable computational time. Moreover, MD simulations systems are well-prepared by applying various tools, which, for instance, constraint covalent bonding between hydrogens and heavy atoms, smooth long-range electrostatic interactions, and add all missing hydrogen atoms that were not present in the crystal structure.

While performing MD simulations in a solute environment, the way in which the solvent is perceived plays a crucial role, especially if the studied molecules are able to form multiple hydrogen bonds, such as CDs. There are two approaches to treat water: either implicitly [51] or explicitly [52]. The former is often also called a continuum approach, as it does not distinguish the separate solvent molecules. The most widely used implicit water models are the COnductor-like Screening MOdel (COSMO) [53], Polarizable Continuum Model (PCM) [54], and Generalized Born Implicit Solvent (GBIS) [55]. These calculations are relatively computationally efficient but they deliver less accurate data when it comes to the local solvent density fluctuations around a solute molecule. In comparison to other MM-treated systems, such as ligand–protein systems, CD complexes are relatively small, so a higher level of calculation accuracy is desired and affordable. Therefore, in the vast majority of cases, for MD simulations of CD complexes, the explicit water approach is applied. This means that a definite number of water molecules can be added to the simulation box and the solvent is no longer treated as a continuum. The most widely applied explicit water models are single point charge (SPC) [56] and the three-site model (TIP3P) [56]. However, it should be emphasized that these models need proper fitting and parametrization. Moreover, the water model should be compatible with the applied FF. For example, AMBER FFs are parameterized from gas-phase QM calculations that do not involve any co-parametrization with any water models and, thus, can be used with most of the generally applicable water models, such as TIP3P and SPC.

A significant advancement in this topic is the development of polarizable FFs, such as AMOEBA FF [57].

Implicit approaches were used, but only in a small amount of cases: among the 104 papers on the MD simulations of CD complexes, only seven included an implicit solvation (see Table 2). Implicit solvent models were developed for the QM calculations, which could have prevented their application to MD simulations. One interesting approach was represented in an article about the CD–sertraline complex [45], where, first, the MD output was optimized with the QM method DFT/PBE0/6-31G(d,p)., and, second, binding energies were calculated by performing single-point calculations with the COSMO model. A similar case was reported for the complex of CD with ginsenosides [58]. There, similarly, the last MD nanoseconds were minimized with the COSMO water model, this time with the PM6 semi-empirical method, and from such a system, the binding free energy was calculated. Such an approach could be an alternative to the free energy calculations described below.

### 2.4. Post-MD Simulation Analysis: GBSA, PBSA

MMGBSA is an element needed to estimate the binding free energy between two states: bounded (e.g., CD complex) and non-bounded (e.g., separated CD and guest) in a solution [59]. A basic binding free energy Equation (1) is as follows:

ΔG binding = G complex − [G protein + G ligand](1)

Δ*G* binding can also be understood as an energy composed out of the molecular mechanics energy of binding, and an entropy component (Equation (2)).

Δ*G* binding = Δ*E*_MM_ − TΔ*S* (2)

In MD simulations of a CD-guest system, the entropic contribution can be calculated based on the MD trajectories; however, it is usually omitted. If present, it is then mostly configurational and not a thermal entropy. Configurational entropy can be estimated from trajectory based on the variance–covariance matrix of the atomic positional fluctuations, using a quasi-harmonic method, where the variance–covariance matrix is calculated for all atoms in the complex. In the quasi-harmonic method using Cartesian coordinates, the mass-weighted variance–covariance matrix is first calculated from MD trajectories, in which the overall translations and rotations of the solute molecule are removed using least-square fits of mass-weighted coordinates. For examples, see the section about CD complexes with plant-derived substances (Section 3.5).

In the liquid state, additionally, the solution input must be included in the form of a free solvation energy (Equation (3))

ΔG binding = ΔE MM − TΔS + ΔG sol(3)

It is composed of two elements: electrostatic and non-polar free energies of solvation (Equation (4)). The first is calculated via MM-PBSA (Poisson-Boltzmann Surface Area) or MM-GBSA (Generalized-Born Surface Area), the second is gained by evaluating the solvent accessible area (SASA) [60].

Δ*G* sol = Δ*G* sol-ele + Δ*G* sol-nonpolar (4)

For MMGBSA calculations, the last snapshots of an MD simulation are used. Which snapshots should be considered is determined by the root-mean-square deviation (RMSD) values, as only stabilized systems can be taken into account. RMSD graphs are obtained via a visualization tool, in most of cases it is Visual Molecular Dynamics (VMD) [61]. 

It is possible that the MM-GBSA/PBSA binding free energies might be overestimated due to the application of MM calculations. To correct this, sometimes more precise QM calculations are performed on the last MD snapshots. Energies obtained in this way are called QM-GBSA/PBSA [62]. Examples among CD complexes are found in references [63,64,65].

Another approach (even if not very common in CD calculations) to “precise” the MM-GBSA MD simulation-derived results is via the application of the hybrid ONIOM method [66,67]. It divides the analyzed system into layers treated with different levels of theory. For example, in [66], three layers were defined, namely: chalcones (guests), CD (host), and solvent molecules (water), and optimized with B3LYP/6-31+G(d,p), B3LYP/3-21G, and UFF approaches, respectively.

### 2.5. Umbrella Sampling (US) and Steered (Biased) MD

While performing a classical (unbiased) MD, the analyzed system is trapped in a local minimum. No other local minima in the potential energy surface (PES) were explored, because to do so, an extensively long MD must have been performed [68,69]. As a result, no transition state could be observed. In order to sample more of the PES, an external force must be applied to drag a system over the energy barrier to another local minimum. For that purpose, umbrella sampling (US) was used [68,69]. It drives the system from one thermodynamic state to another; hence, the method’s name (it connects the energetically separated regions). This approach applies an external time-dependent force (bias) to the analyzed system. The system is moved along a predefined reaction’s coordinate. In other words: the sampling region is constrained by a biasing potential, which allows a host and a guest to sample this configurational space. The other mean for the calculation of the equilibrium free energy, from a large set of independent (and not necessarily equilibrium) simulations, is by using the Jarzynski equality [68]. This equation relates free energy differences between two states and the irreversible work along an ensemble of trajectories joining the same states.

In the case of US, the transition over an energy barrier is simulated by freezing (restraining) the reaction’s coordinate for a given time, called a ‘window’. As Kästner explains in the ‘Umbrella Sampling Review’: “The force on the frozen reaction coordinates is sampled. The resulting mean force is the derivative of the free energy with respect to the reaction coordinate. Integration of the mean force results in the potential of mean force (PMF)” [70].

A whole US process is composed of several ‘windows’, which are distributed in such a manner that they overlap, which results in a full analysis of the reaction coordinate. At each window, a classical MD simulation is performed. Consequently, at each window (so: for each configuration), a binding free energy in a form of PMF is calculated. In CD systems, the reaction coordinate is often defined as the center of mass (COM) distance, which is a space between the COM of the CD and a guest. More precisely, PMF of each window is plotted against the COM distance. The resulting final diagrams show the relationship between Δ PMF and Δ*G* (binding free energy). Generally, in this approach, Δ*G* is a function of a certain coordinate. After the simulations, the free energy profile along the pathway can be reconstructed using a post-processing weighted histogram analysis method (WHAM) [71].

If the bias potential is varied, in regard to pulling the system from one state to another, rather than having a changing fixed bias for the particular steps, and if this variation is slow when compared to the relaxation of the system, the whole approach is called steered (biased) MD (SMD) [68,69]. In contrast to classical MD, which cannot deliver accurate results of guest-release from CD, SMD serves as a method to analyze this transition state.

Published US and/or SMD applications for CD–guest systems, including technical aspects, such as window simulation times, were presented in Table 2: I.F.

### 2.6. Coarse-Grained MD

Coarse-grained (CG) MD are quite different from the above-mentioned all-atom MD simulations. In the CG MD approach, molecules are not represented by individual atoms, but by coarse-grained sites, approximating groups of atoms, such as whole amino acid residue. By decreasing the degrees of freedom, much longer simulation times can be studied at the expense of molecular details [72].

There are few examples of the CG MD simulations in the studies on CD complexes. In these few, it was applied to investigate either a typical host–guest interaction or a more complex membrane-including system. One case included β-CD and adamantane [73]. In this work, the CD structure was represented by two bead types: hydrophobic and hydrophilic. Application of the CG MD was, here, mainly caused by the fact that, in this particular study, a far larger structure was a goal, namely a multiblock copolymer, including adamantane and CD molecules.

The other two examples correspond to the membrane-including systems. In [74], the inclusion of the cholesterol molecule into the CD was analyzed with the simultaneous CD-dimers adsorption on the membrane/water interface. In this study, the CG MD approach was applied to test the prediction that cholesterol can be more easily extracted from a liquid-disordered phase. The CG systems include the planar lipid bilayer and a small liposome.

Another example is a work describing the binding of small functionalized dendrimer molecules to β-CD-terminated self-assembled monolayers [75]. The formerly performed all-atom MD simulations on the same system were used as a basis for the usage of the longer timescales in the CG MD approach.

It is interesting to note that a similar idea appeared a decade later. Škvára and Nezbeda, in their work published in 2018 [76], stated that they performed all-atom MD simulations of methanol systems, including racemic ibuprofen and β-CD, in order to use those results for later development of a CG model.

## 3. Application of the Molecular Dynamics Simulations for Systems Including Cyclodextrins—The Most Important and Interesting Cases

We extracted the important information on the MD simulations of CDs, and their complexes, from articles published in the last 20 years, and present the information in Table 2. In some cases, crucial data concerning the equilibrium run is missing, due to insufficient descriptions of the methods in the cited articles. No substantial articles on this topic were found prior to 2012. Previously, for the most part, simple molecular docking and Monte Carlo simulations were performed. It was described in the review by Quevedo and Zoppi [16]. The mentioned review is by no means concentrated on the MD calculations; it covers up until 2016, whereas the most important changes in the MD simulations of CDs took place in the last 5 years, which is roughly the period of 2016–2021.

Two main purposes for CDs application in the research have emerged: (i) CDs as drug carriers and (ii) CDs as extracting agents. As this article is meant to provide guidance for those wanting to perform MD simulations of CDs, we constructed this review from the applicability perspective of CDs.

### 3.1. CDs Used as Drug Carriers (Water Environment)

There is a wide range of molecules that have been modeled in CD systems using the MD approach. It would be difficult to divide these diverse examples into groups based on their chemical structures. Therefore, a less chemical (but a more activity-based) division was imposed. The published cases were gathered in the following groups: non-steroidal anti-inflammatory drugs (NSAIDs), anti-fungal drugs and antibiotics, plant-derived substances, and others. The last group includes all substances (mostly drugs) that could not be classified to any of the abovementioned categories. A separate subsection has been dedicated for the articles that apply umbrella sampling or/and SMD, and for those that concern a complex stoichiometry other than a 1:1 CD.

As in many works, the general conclusions derived from the research are, in principle, very similar, not all of the cases have been thoroughly described in the text. The repetitive findings are: 

Among native CDs, apart from big guests, such as antibiotics, the most stable complexes are mostly formed with β-CD, which is also in agreement with experimental results.

Among substituted CDs, HP-β-CD forms the complexes characterized by the highest binding affinity, in most cases.

The main driving force of the guest inclusion in CDs are vdW interactions.

H-bonding is the main factor stabilizing the CD complexes.

Data obtained from MD simulations are consistent with data from the experiments, both in terms of structural and binding properties.

Such reoccurring results, as well as the number of articles on the MD–CD topic, emphasize the usefulness of this calculation technique, in terms of CD analysis.

**Table 2:** If not specified, the pressure in the equilibrium is the same as in the production run. ‘no i.p.’ stands for ‘no information provided’. Equilibration does not include the geometry optimization (reaching geometry equilibrium) and solvent molecule relaxation. Regarding production run, if not specified, otherwise: NVT ensemble. * See also: ibuprofen racemic mixture in part II of the table. ‘Standard parametrization procedures’, as mentioned in the table, are described in Section 2.2.

N°	Reference	CD	Guest	Software Used for MD	Force Field	Equilibration Time and Conditions	Production Run Time and Conditions	Time Step	Water Model	Temperature (K)
I. CDs used as drug carriers (water environment)										
A. 2:1 host–guest CD complexes										
1	[77]	β-CD	piroxicam	Materials Studio and Insight II/Discover packages	CVFF	no i.p.	10 ns (1:1 host–guest), 100 ns (2:1 host–guest)	1 fs	implicit	300
2	[78]	β-CD	posaconazole	GROMACS	GROMOS53A6	no i.p.	100 ns, NPT (*p* = 1 bar)	no i.p.	implicit; water and hydrogen peroxide	298
3	[79]	β- and HP-β-CD	sulfamethoxazole	Desmond	OPLS2005	1 ns	15–20 ns, NPT (*p* = 1 bar)	no i.p.	TIP3P	300
4	[80]	β-CD	17-α-methyltestosterone	AMBER 12	Glycam06h, GAFF	200 ps NVT, 200 ps NPT	50 ns, NPT (*p* = 1 bar)	no i.p.	TIP3P	298
5	[81]	α-, β-, 2,6-DM-β-, and 2,3,6-TM-β-CD	citral isomers	AMBER 12	Clycam06 (native CD), q4md-CD (CD-derivatives), GAFF (guest)	no i.p.	12 ns	1 fs	TIP3P	300
6	[82]	2-HP-β- and 2-HP-γ-CD	imazapyr	Desmond	OPLS2005	no i.p.	30 ns, NPT (*p* = 1 atm)		TIP3P	300
B. NSAIDs*										
1	[83]	HP-β-CD	etodolac and L-arginine	Desmond	OPLS2005	no i.p.	5 ns, NPT (*p* = 1.013 bar)	2 fs	TIP4P	300
2	[84]	β- and HP-β-CD derivatives	flurbiprofen, ibuprofen, ketoprofen, and naproxen	GROMACS	ffgmx (derivative of GROMOS87)	no i.p.	500 ps	no i.p.	no i.p.	300
3	[85]	β-CD	ketoprofen	GROMACS	GROMOS	20 ps	10 ns, 20 ns, NVT	2 fs	no i.p.	298
4	[86]	α-, β-, γ-, HP-β-, M-β-, and SBE-β-CD	ketoprofen	AMBER 14	GAFF	NVT, NPT	100 ns, NPT (*p* = 1 bar)	2 fs	TIP3P	310
5	[87]	SBE-β-CD	celecoxib	YASARA	AMBER ff14SB	no i.p.	600 ns	1.25 (intramolecular forces), 2.5 fs (intermolecular forces)	explicit	298
6	[88]	α-, β-, and γ-CD	etoricoxib	AMBER 11	Glycam06h (CD), GAFF (guest)	60 ps NVT, 1000 ps NPT	20 ns, NPT (*p* = 1 bar)		TIP3P	298
7	[89]	α-, β-, and γ-CD	nabumetone	AMBER 14	GLYCAM-06j (CD), GAFF (guest)	120 ps NVT, 2 ns NPT	5 µs, NVT	2 fs	TIP3P	300
8	[90]	β-CD	*R*- and *S*-ketoprofen	Desmond	OPLS2005	12 ps NVT (10 K), 12 ps NPT (10 K), 24 ps NPT (300 Km 1 atm), 24 ps (300 K, 1 atm)	50 ns, NPT (*p* = 1.01325 bar)	no i.p.	TIP4P	300
9	[91]	α-, β-, and γ-CD	antipyrine	AMBER 12	FF99SB	50 ps NVT, 500 ps NPT	10 ns, NPT (*p* = 1 bar)	no i.p.	TIP3P	300
C. Anti-fungal drugs and antibiotics										
1	[92]	2,6-DM-β-CD	natamycin	GROMACS	GROMOS96	no i.p.	30 ns, NPT (*p* = 1 bar)	no i.p.	TIP3P	300
2	[93]	α-, β-, γ-, and 2-HP-β-CD	cefuroxime axetil	GROMACS	GROMOS 56A6	1 ns NPT	500 ns NPT (*p* = 1 bar)	2 fs	SPC	298
3	[94]	γ-CD	alamethicin	CHARMM	CHARMM36	5 ns NVT	1000 ns, NPT (*p* = 1 atm)	no i.p.	TIP3P	303
4	[95]	α-, β-, and γ-CD	chloramphenicol	AMBER 14	no i.p.	heating up to 300 K, 50 ps; NVT 500 ps	10 ns, NPT (*p* = 1 bar)	no i.p.	TIP3P	300
5	[96]	β- and γ-CD	amphotericin B	NAMD	CSFF, CHARMM27	no i.p.	10 ns	2 fs (short-range interactions), 4 fs (long-range interactions)	TIP3P	300
D. Plant-derived substances										
1	[97]	β-, 2-HP-β-, 6-HP-β-, 2,6-DHP-β-, 2,6-DM-β-, and RM-β-CD	2-acetyl-1-pyrroline	AMBER 16	Glycam06 (CD), GAFF2 (guest)	500 ps (heating up)	500 ns, NPT (*p* = 1 atm)	2 fs	TIP3P	298
2	[98]	β- and γ-CD	polydatin	AMBER 14	GAFF	200 ps (heating up), 300 ps NVT	55 ns, NPT (*p* = 1 bar)	2 fs	TIP3P, 2545 ±29 water molecules	300
3	[99]	γ-CD	3-hydroxyflavone	AMBER 16	Glycam06 (CD), GAFF (guest)	100 ps NVT	300 ns, NPT (*p* = 1 atm)	no i.p.	TIP3P	298
4	[100]	β- and HP-β-CD	borneol	GROMACS	GROMOS54a7	NVT, NPT (2 fs time step)	100 ns, NPT	1 fs	no i.p.	300
5	[101]	β-, 2,6-DM-β-, 2-HP-β-, 6-HP-β-, and 2,6-DHP-β-CD	eucalyptol	AMBER 14	Glycam06-h (CD), GAFF (guest)	100 ns, NVT	70 ns NPT (*p* = 1 atm)	2 fs	SPC, 2000 water molecules	298
6	[102]	β- and γ-CD	triterpene glycoside and glycyrrhizic acid	PRESTO	GAFF	10,000 steps (heating up), 200,000 NVT	0.8 ns	1 fs	TIP3P	300
7	[103]	β-, 2,6-DM-β-, 2-HP-β-, 6-HP-β-, 2,6-DHP-β-, and RM-β-CD	luteolin and pinocembrin	AMBER 16	Glycam-06 (CD), GAFF (guest)	60 ps (heating up)	100 ns, NPT (*p* = 1 atm)	2 fs	TIP3P	298
8	[104]	β-, 2,6-DM-β-, and HP-β-CD	mansonone G	AMBER 16	Glycam-06 (CD), GAFF (guest)	60 ps (heating up)	90 ns, NPT (*p* = 1 atm)	2 fs	TIP3P	303
9	[105]	β-, 2,6-DM-β-, 2-HP-β-, 6-HP-β-, and 2,6-DHP-β-CD	pinostrobin	AMBER 12	Glycam06 (CD), partial charges of guest: standard parametrization procedures	100 ps (heating up)	80 ns	2 fs	explicit, 1400+- 42 water molecules	298
10	[64]	β-, 2,6-DM-β-, DM-β-, and randomly methylated β-CD	hesperetin and naringenin	AMBER 12	Glycam06 (CD), partial charges of guest: standard parametrization procedures	100 ps (heating up)	80 ns, NPT (*p* = 1 atm)	2 fs	SPC	298
11	[65]	β- and 2,6-DM-β-CD	naringenin	AMBER 12	Glycam06 (CD), partial charges of guest: standard parametrization procedures	100 ps	80 ns, NPT	2 fs	SPC, 1480 ± 10 and 1750 ± 3 water molecules	298
12	[106]	β-CD	daidzin	GROMACS	GROMOS96	NPT	12 ns, NPT (*p* = 1 atm)	0.002 ps	explicit, 3100 water molecules	300
13	[107]	γ-CD	glycyrrhizin	CHARMM	added from cff	no i.p.	1 ns	1 fs	explicit, 2969 water molecules for β-CD and 5718 for γ-CD	300
14	[108]	β-CD	eriocitrin (flavanone)	AMBER 19	Glycam06j (carbohydrates, 2-hydroxypropyl units), missing parameters and atom types: GAFF2	1 ns (heating and cooling: 0 K <-> 300 K); 5 ns, NPT	200 ns, NVT	2 ps	explicit, 8527–9303 water molecules	300
15	[109]	α-, β-, and γ-CD	cannabidiol	GROMACS	OPLS-AA	no i.p.	250 ns, NPT (*p* = 1 bar)	2 ps	no i.p.	298, 310, 322, 334
16	[110]	β- and γ-CD	rosmarinic acid	AMBER	q4md-CD (CD), GAFF (guest)	no i.p.	50 ns, NPT	no i.p.	TIP3P	300
17	[111]	β-CD	harman (alkaloid)	GROMACS	GROMOS 54a7	NVT, NPT, 100 ps	50 ns, NPT (*p* = 1 bar)	2 fs	SPC	300
18	[112]	β,- γ-, HP-β-, and DM-β-CD	myricetin	Desmond 2018.4	OPLS3	no i.p.	30 ns, NPT (*p* = 1.013 bar)	no i.p.	TIP3P	300
19	[113]	HP-β-CD	capsaicin	AMBER 16	GAFF	NVT 50 ps, NPT 50 ps	5000 ps, NPT (*p* = 1 atm)	2 fs	TIP3P	300
20	[58]	β- and γ-CD	pseudoginsenoside PF11	YASARA	AMBER 14	no i.p.	100/150 ns, NPT (*p* = 1 bar)	no i.p.	COSMO	298
21	[114]	α- and β-CD	thymol	Desmond	OPLS 2005	no i.p.	48 ns	no i.p.	SPC	
22	[115]	α-, β-, and γ-CD	daidzein (isoflavone)	AMBER 12	q4md-CD (CD), GAFF (guest)	no i.p.	50 ns, NPT (*p* = 1 atm)	no i.p.	TIP3P	300
23	[116]	α-, β-, and γ-CD	cathinone	AMBER	GAFF	200 ps NVT, 20000 ps NPT	30 ns, NPT (*p* = 1 bar)	2 fs	TIP3P	298
24	[117]	2,6-DM-β- and 2,3,6-TM-β-CD	β-citronellol	AMBER	CLYCAM (β-CD), q4md-CD (methylated β-CD)	NVT, 250 ps NPT	11 ns, NPT	no i.p.	explicit	300
25	[118]	β- and HP-β-CD	naringin	AMBER 14	GAFF	no i.p.	100 ps, NPT (*p* = 1 bar)	no i.p.	TIP3P	no i.p.
26	[119]	γ- and HP-γ-CD	naringin	Desmond	OPLS2005	no i.p.	100 ps, NPT (*p* = 1.0325 bar)	2 fs	VSGB 2.0 (implicit)	310
27	[120]	2-HP-β-CD	quercetin	AMBER 14	GLYCAM_06j-1 (CD part of molecule), GAFF (2-HP groups of CD and guest)	no i.p.	400 ns	no i.p.	TIP3P	300
28	[121]	HP-β-CD	silibinin	AMBER 12	GLYCAM_06i-12SB (CD-part of molecule), GAFF (2-HP groups of CD)	100 ps NVT, 100 ps NPT	190 ns and 250 ns	no i.p.	TIP3P, 3841 water molecules	300
29	[122]	β-CD	cyanidin-3-*O*-glucoside	AMBER 10	GLYCAM_04 (CD), GAFF (guest)	100 ps NPT	30 ns, NPT	2 fs	TIP3P	303.15
30	[123]	β-CD	resveratrol	AMBER 11	GLYCAM_06 (CD), GAFF (guest)	100 ps NVT	20 ns, NPT (*p* = 1 atm)	2 fs	no i.p.	300
31,32	[124,125]	β-, 2,6-DM-β-, and 2-HP-β-CD	α-mangostin	AMBER (PMEMD module)	Glycam06j (CD)	10 ns NVT	100 ns, NPT (*p* = 1 atm)	2 fs	TIP3P	298
E. Others										
1	[126]	3-mono-amino-β-LHRH (luteinizing hormone releasing hormone) conjugate		MacroModel (implicit water model), Desmond (explicit water model)	OPLS2005	1 ns for implicit water model	20 ns for implicit water model; 40 ns, NPT (*p* = 1.01325 bar) for explicit water model	2 fs (explicit water model)	implicit and explicit (SPC, 2618 water molecules)	298.1
2	[127]	HP-β-CD	efavirenz and L-arginine	Desmond	OPLS2005	no i.p.	5 ns, NPT (*p* = 1.013 bar)		TIP4P	300
3	[128]	β- and M-β-CD	omeprazole and L-arginine	GROMACS	ffgmx	3 ns	15 ns (L-arginine: drug, 1:1), 6 ns (other L-arginine-drug ratio), NPT	1.5 fs	explicit, more than 1000 water molecules	300
4	[129]	β- and γ-CD	pyrazoline dye	MOPAC2012	Amber99	50 ps NVT, 2000 ps NPT	2000 ps	2 fs	TIP3P	298
5	[130]	β-CD	cyanine dye	SYBYL-X	Tripos	500 fs per each 20 K gain; then 25 ps NVT	2 ns	2 fs	Molecular Silverware algorithm	300
6	[131]	β-CD	carbazole-based dyes	Chem3D Pro	MM2	no i.p.	no i.p.	2 fs	no i.p.	no i.p.
7	[132]	α-, β-, γ-, and 6-HP-β-CD	lutein	AMBER 14	GAFF	10000 steps (heating up)	100 ns, NPT (*p* = 1 bar)	2 fs	TIP3P	310
8	[133]	β-CD	maltogenic amylase	GROMACS	GROMOS96	50 ns	10 ns	2 fs	SPC	343
9	[134]	sulfated β- and M-β-CD	levosulpiride	AMBER 9	GAFF	10 ps (heating up)	3 ns	2 fs		300
10	[135]	HP-β- and 2,6-DM-β-CD	bisacodyl	Forcite	COMPASS	50 ps, 298 K, NVT	40 ps	1 fs	explicit, 20 water molecules	500 → 300
11	[136]	α-, β-, γ-, and differently substituted β- and γ-CD	chlorpromazine	Amber 16	q4md-CD (CD), GAFF (guest)	heating up by 25 ps periods, 200 ps relaxation	50 ns, NPT (*p* = 1 atm)	2 fs	TIP3P	300
12	[137]	α- and β-CD	ambroxol hydrochloride	MOE	MMFF94x.	100 ps	500 ps	no i.p.	no i.p.	300
13	[45]	β- and 2-HP-β-CD	sertraline	Tinker code v8.4	MM3	no i.p.	no i.p.	no i.p.	COSMO	298
14	[138]	γ-, HP-γ-, and HP17-γ-CD	lopinavir	GROMACS	GROMOS-96 54a7	NVT (1 ns, 300 K), NPT (2 ps, 300 K, 1 bar)	100 ns, NPT (*p* = 1 bar)	no i.p.	SPC	300
15	[113]	β-, γ-, HP-β-, and M-β-CD	glipizide	AMBER 14	GAFF	no i.p.	70 ns	2 fs	TIP3P	310.15
16	[139]	β-CD	metyrapone	YASARA	AMBER14	no i.p.	136 ns, NPT (*p* = 1 bar)	no i.p.	explicit	298
17	[140]	β-CD	calixarene sulfonates with 4-aminoazobenzene	LAMMPS	AMBER	1 ns	20 ns, NPT (*p* = 1 bar)	2 fs	TIP4P2005, 2000 water molecules	300
18	[141]	2,3,6-TM-β-CD	temoporfin	AMBER 14	q4md-CD (CD), GAFF (guest)	500 ps, NPT	10 ns, NPT (*p* = 1 atm)	1 fs	TIP3P	300
19	[142]	β-CD	theophylline	GROMACS	amber99sbildn	0.1 ns NVT, 1 ns NPT	50 ns	no i.p.	TIP3P, 1353 water molecules	300
20	[143]	2,6-DM-β- and 2,3,6-TM-β-CD	α-naphthaleneacetic acid	AMBER 12	q4md-CD (CD), GAFF (guest)	250 ps NPT	11 ns, NPT	no i.p.	TIP3P	300
21	[144]	β-, γ-, and randomly sulfated β- and 6-S-β-CD	medetomidine	AMBER	parm10 and ff14SB	120 ns	100 ns, NPT (*p* = 1 atm)	2 fs	explicit	300
22	[145]	β-, DM-β-, TM-β-, and randomly methylated β-CD	glycocholate	AMBER 12	GAFF, q4md-CD (CD-derivatives)	400 ps (heating up)	2 ns, NPT (*p* = 1 atm)	2 fs	TIP3P	300
23	[146]	β- and 2,3-di-*O*-acetyl-β-CD	clenpenterol	no i.p.	Amber	40 ns	100 ns	no i.p.	explicit	no i.p.
24	[147]	β-CD	norepinephrine	Desmond	OPLS2005	no i.p.	15 ns, NPT (*p* = 1bar)	no i.p.	TIP3P	300
25	[67]	RM-β- and HP-β-CD	triamcinolone	GROMACS	GROMOS 54a7	NVT 5 ns, NPT 5 ns	200 ns	no i.p.	TIP3P	298
26	[148]	HP-β-CD	1-indanone thiosemicarbazones	GROMACS	GROMOS96 53a6	no i.p.	100 ns	1 fs	SPC	300
27	[149]	α-, β-, γ-, and 2-HP-β-CD	fentanyl	AMBER	literature source (CD), GAFF (guest)	200 ns (heating up), 2.5 ns (equilibrium)	10 ns or 30 ns (depending on guest)	2 fs	TIP3P	300
28	[150]	HP-β-CD	clonidine	GROMACS	GROMOS-96 53a6	no i.p.	100 ns	1 fs	SPC	300
29	[151]	sulfobutylether-β-, sulfated β-, and monochlorotriazinyl-β-CD	propiconazole nitrate	GROMACS	q4-MD (CD), GAFF (guest)	no i.p.	50 ns, NPT (*p* = 1 atm)	no i.p.	TIP3P	293,15
30	[152]	β-CD	mammea A/AA	AMBER 12	ff99SB	no i.p.	10 ns, NPT (*p* = 1 atm)	2 fs	TIP3P, 1452 water molecules	300
31	[153]	HP-β-CD	carbamazepine	NAMD	CHARMM	5 ps heating up, 50 ps equilibration	2 ns	1 fs for covalent, 2 fs for vdW, 4 fs for electrostatic atom interactions	no i.p.	300
32	[154]	β-CD	methotrexate	AMBER 12	GLYCAM_06 (CD), GAFF (guest)	1 ns	10 ns, NPT	2 fs	TIP3P	298
33	[155]	α-, β-, and γ-CD	cumene hydroperoxide	GROMACS	GROMOS96	no i.p.	16 ns	no i.p.		298
34	[156]	β-CD	*N*-methyl carbamates	AMBER 7	GAFF	no i.p.	3 ns, NPT (*p* = 1 atm)	2 fs	TIP3P, 9000 water molecules	300
35	[157]	β- and randomly methylated β-CD	isosorbide diesters	AMBER 10	GAFF	no i.p.	10 ns, NPT (*p* = 1 bar)	2 fs	TIP3P	300
36	[158]	β-CD	adamantane derivatives	NAMD	CHARMM (β-CD), CGenFF program (bond, angle and dihedral parameters of guest) and CHARMM GFF (atomic charges of guest)	10 ns	20 ns	2 fs	TIP3P	no i.p.
37	[159]	amino-β-CD (protonated and non-protonated)	doxorubicin	NAMD	CHARMM FF	no i.p.	30 ns, NPT (*p* = 1 atm)	0.5 fs	TIP3P	298.15
38	[160]	β-CD	caffeine	GROMACS	GROMOS 56A	no i.p.	4000 ps	1 fs	explicit, 1000 water molecules	333.15
39	[161]	β-CD	zwitterionic phenylalanine	PINY-MD code	GROMOS	NVT; NPT (*p* = 0 bar) 500 ps	30 ns, NVT	4 fs	explicit, 2903 water molecules	300
F. Umbrella sampling and steered (biased) molecular dynamics										
1	[162]	β-CD	trimethylammonium adamantane salt	AMBER 16	Glycam06 (CD), GAFF2 (guest)	unbiased MD: 100 ps (1 fs time step, heating up); 500 ps NVT density equilibration; NPT	NPT (*p* = 100 kPa); SMD	1 μs (unbiased MD), 4.4 μs (biased MD)	TIP3P	300
2	[163]	β-CD	adamantane-doxorubicin	GROMACS	AMBER99SB-ILDN	NVT 2 ns, NPT 2 ns	100 ns, NPT (*p* = 1 bar); umbrella sampling: 10 ns	no i.p.	TIP3P, 15,000 water molecules (for 6 drugs in one water box)	310
3	[164]	α-, β-, and γ-CD	adamantane-terminated gold nanoclusters	NAMD	potential model based on CHARMM (CD, guest), GolP (interactions with gold atoms)	no. i.p.	10 ns, NPT (*p* = 1 atm); SMD; umbrella sampling for each window: 500 ps	2 fs	SPC	300
4	[165]	2-HP-β-CD	cilexetil	GROMACS	MMFF (guest), CHARMM (water)	NVT, NPT	umbrella sampling for each window: 500 ps	2 fs	no i.p.	310
5	[66]	β-CD	chalcone and 2′,4′-dihydroxychalcone	GROMACS	GROMOS 53a6	10 ns for each window	100 ns for each window, total time: 4290 ns	2 ps	SPC water model, 1200 water molecules	310
6	[63]	β-CD	genistein	AMBER, umbrella sampling: GROMACS; DFTB-MD	Glycam06 (CD), partial atomic charges of guest: standard parameterization procedures	2 ns (SMD)	unbiased MD: 100 ns, NPT (*p* = 1 atm); SMD: 8 ns; DFTB+: 1000 ps	2 fs, DFTB+: 1 fs	SPC, 1400 water molecules	298, DFTB+: 400
7	[166]	β-, 2,6-DM-β-, and 2-HP-β-CD	pinostrobin	GROMACS	Glycam06 (CD), GAFF (guest)	no i.p.	1 ns, NPT	no i.p.	SPC, 3200 water molecules	289
8	[167]	β-CD	cinnamaldehyde and eugenol	NAMD 2.6	Charmm33b	50 ps heating up	1.2 ns	no i.p.	no i.p.	298
9	[168]	α-, β-, and γ-CD	umbelliferone	GROMACS	q4md (CD), GAFF (solvents, guest)	2 ns	NPT (*p* = 1 bar); total time: 400 ns; 10 ns for each window	no i.p.	water (TIP3P model, 2:1 complex); other solvents (2:2 complex): methanol, ethanol, dimethyl sulfoxide, *N*,*N*-dimethylacetamide, *N*,*N*-dimethylformamide, acetone, tetrahydrofuran, acetonitrile, chloroform	300
10	[69]	β-CD	1-butanol and aspirin	AMBER 14	no i.p.	umbrella sampling: 1 ns NVT; heating up at 200 K, 250 K, 298 K for 150 ps	100 ns: conventional MD; 10 ns: SMD (NPT, *p* = 1 bar); 2.5 ns: umbrella sampling	no i.p.	TIP3P	300 (SMD), 298 (umbrella sampling)
II. CDs used as extracting agents (different solvents)										
1	[169]	β-CD	2,3,7,8-tetrachlorodibenzo-*p*-dioxin	GROMACS	GROMOS96	no i.p.	overall 12 ns (equilibrium + run), NPT (*p* = 1 bar)	0.001 ps	SPC, 2500 water molecules	298
2	[170]	α-, β-, and γ-CD	2,2′,5,5′-tetrachlorobiphenyl	Discover Model of Materials Studio	COMPASS	no i.p.	NPT (*p* = 1 atm)	0.5 fs	COSMO, 800 water molecules	298
3	[171]	α-, β-, and γ-CD	DDT	NAMD	CHARMM27	no i.p.	NPT (*p* = 1 atm)	2 fs	TIP3P	298
4	[172]	quaternary ammonium β-CD	ochratoxin A	HyperChem	Amber	no i.p.	a few hundreds of ps time		TIP3P	298
5	[173]	β-CD	PCB126	GROMACS	GROMOS96	no i.p.	15 ns, NPT (*p* = 1 bar)	1 fs	SPC, 2500 water molecules	300
6	[174]	β-CD	ibuprofen (racemic mixture)	GROMACS	GROMOS54a7	1 ns NVT, 10 ns NPT (P = 1 bar)	100 ns, NVT	1 fs	methanol, 2000 molecules	260–380
7	[76]	β-CD	ibuprofen (racemic mixture)	GROMACS	GROMOS54a7	1000 ps NPT (P = 1 bar), 500 ps NVT	10 ns	1 fs	methanol, 2000 molecules	273.15
8	[175]	HP-β-CD	(*E*)-piceatannol	GROMACS	GROMOS53A6	no i.p.	2 ns, NPT (*p* = 1 atm)	2 fs	SPC/E (water), methanol + water, ethanol + water, n-propanol + water, glycerol + water cosolvents	298.2
9	[176]	β-, 2,6-DM-β-, and 2-HP-β-CD	UC781	AMBER 10	Amber parm03	400 ps NVT, 11 ns NPT	10 ns, NPT (*p* = 1 atm)	2 fs	308 water molecules (TIP3P), 1670 ethanol molecules	300
10	[177]	γ-CD	gold nanoparticles	GROMACS	CHARMM36	100 ps; NPT 100 ps	200 ns, 250 ns (depending on the number of CD molecules), NPT (*p* = 1 bar)	2 fs	explicit, 10,500–12,620 water molecules	298.15
11	[178]	β- and 2,3-di-*O*-acetyl-β-CD	terbutaline enantiomers	AMBER 12	q4md-CD and Glycam04 and Amber99SB (CD), GAFF (guest molecules with one positive charge)	400 ps NVT	6 ns, NPT (*p* = 1 atm)	2 fs	TIP3P	300
12	[179]	γ-CD	regioisomers of bis-*N*-methylfulleropyrrolidines	NAMD	CHARMM	20 ns	60 ns, NPT (*p* = 1 bar)	2 fs	DMSO, water (TIP3P)	298
13	[180]	permethylated β-CD	phenylazetidin derivatives	GROMACS	GROMOS	no i.p.	4 ns, NPT (*p* = 1 bar)	no i.p.	no i.p.	300
14	[181]	β-CD	isoleucine enantiomers	no i.p.	AMBER ff99SB	no i.p.	5 ns	1 fs	2021	293
15	[182]	β-CD	terminally blocked phenylalanine dipeptide (Ace-Phe-Nme),	AMBER 9	Amber 03	no i.p.	8 ns, NPT (*p* = 1 atm)	no i.p.	TIP3P	300

### 3.2. Host–Guest Stoichiometry of CD Complexes

The vast majority of works depict standard systems with one CD molecule and one guest molecule (1:1 host–guest stoichiometry). However, there are a couple of examples of the 2:1 host–guest stoichiometry systems. They are modeled with MD, with reference to the data obtained from the various analytical experiments, such as DSC, PXRD, SCXRD, FT-IR, and NMR, which confirm that the given molecule crystallizes in the 2:1 host–guest stoichiometry. The guests of such complexes mentioned in the literature are: piroxicam [77], posaconazole [78], sulfamethoxazole [79], 17-α-methyltestosterone [80], and citral isomers [81]. Data concerning the MD calculation details of these systems are presented in Table 2: I A. All of these substances have relatively large molecules and exhibit some symmetry, or at least possess similar chemical groups at both ends. 

**Figure 3:**
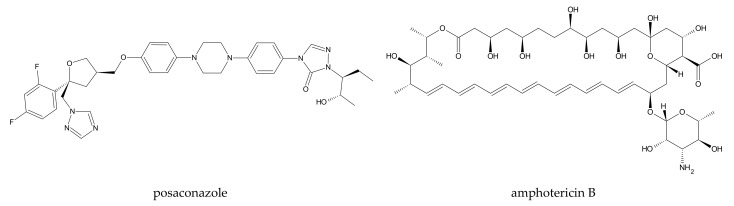
Posaconazole and amphotericin B: antimicrobial drugs complexed with CDs and analyzed with MD simulation. Description in the main text, in Section 3.2 and Section 3.4.

One good example that represents both of these features is posaconazole (Figure 3). This particular case could be used as an industry model for the drug–CD-joined experimental and computational analysis and implementation of research results. The mentioned drug is not only poorly soluble in water, but also sensitive to oxidation. Posaconazole’s encapsulation in CDs lowers the compound’s oxidation rate and, therefore, stabilizes it. This significantly enhances the drug’s half-life and shelf-life time. MD simulation clearly showed, at the molecular level, the reason for this improvement via complexation. At first, the posaconazole–CD complex was simulated in water. Later, the environment was changed into ‘water + hydrogen peroxide’ to simulate the oxidation conditions. The comparison of these two simulations shows that the distance between the drug’s oxidation-sensitive N-atoms and hydrogen peroxide molecules increased in the latter case. This indicates that CDs indeed protect the drug from oxidation by making a kind of mechanical barrier between posaconazole and the oxidative agent. A general take-home message from this research is that, first, we can perform MD simulations, including CDs and molecules, even elongated as posaconazole. Second, it is possible to effectively model an oxidative stress situation.

Another interesting example is work authored by Carvalho et al. [80], where 17-α-methyltestosterone was simulated with β-CD in 1:2 stoichiometry. To the authors’ best knowledge, this is the one CD-incorporated and MD-simulated hormone, thus far. There is one more CD-hormone submitted to the MD calculations, namely LHRH, but it was simulated in the form of a CD-conjugate, not as a CD complex (see, in Table 2: I.E. (CDs used as drug carriers (water environment)—Others), N° 1) [126].

Similarly to the previous case, for the CD-17-α-methyltestosterone, the 2:1 host–guest stoichiometry was determined by the DSC, NMR analysis, and solubility tests. Moreover, it was demonstrated that the complex fraction was 76%. This was displayed by MD simulations as well. First, the system was simulated in vacuum, and the results showed 2:1 host–guest complexation. Afterwards, analogical calculations were run in a water environment. Out of these simulations, a different picture of the complex occurred. Namely, for the first 30 ns out of the 50 ns-long MD simulation, the complex remained in a form of 2:1, with mass centers of the two CD molecules being in a 7.8 Å distance. However, in the next 20 ns, this distance changed to 9.5 Å, and the complex of two CDs and one guest remained half-opened for the rest of the simulation. Therefore, the whole MD-simulated complex was called, ‘pseudo 2:1′. The results correspond with, and explain the experimental 76% complexing fraction.

In all cases gathered in Table 2: A., application of MD simulations reproduced or helped to explain the inner structure of the 2:1 CD complexes, previously determined experimentally.

### 3.3. NSAIDs

NSAIDs are widely used drugs and the works concerning MD analysis of their encapsulation in CDs are among some of the first published in the early 2010s. For data concerning the simulation process, look at Table 2: I.B. In 2013, Suárez and Díaz [89] analyzed the CD fluctuations in water, and after comparing with the β-CD-nabumetone system, they stated that the presence of a drug significantly dampens down the structural fluctuations of the CD ring, resulting in the loss of conformational entropy and, consequently, influencing the total binding energy of the CD complex.

For the last 20 years, various aspects of NSAID complexations with CDs have been taken into account when applying MD simulations. For example, a detailed insight of ketoprofen binding into the β-CD cavity was delivered by Guzzo et al. [85] and Yousef et al. [88], concentrating on α/β/γ-CD-etoricoxib stability, with regards to the changing pH of the environment.

A thorough examination of various CD complexes (α-CD, β-CD, γ-CD, HP-β-CD, M-β-CD, and SBE-β-CD) with ketoprofen was recently conducted [86]. This study encompassed both host–guest stoichiometry, host–guest orientation, and the analysis of interacting forces. For instance, MD simulations revealed that the large γ-CD cavity could host two ketoprofen molecules at once and such a complex remains stable. However, we should note that a 1:1 complex shows higher stability. Similarly, for β-CD, a 1:1 complex is the most stable, but at high concentrations, a 1:2 complex could be formed as well.

### 3.4. Anti-Fungal Drugs and Antibiotics

In the majority of cases, both antifungal and antibacterial compounds have a non-polar character, because they ought to interfere with the lipid layers of their targets. Therefore, their solubility is relatively low. This is why, for those APIs, an appropriate drug carrier enhancing their solubility is desired. One possibility could be encapsulation in CDs. However, these potential guests are, in most cases, large molecules, often in ring-form, or contain numerous non-aromatic rings, which make the simulations more difficult (see the example of amphotericin B, Figure 3).

The case of amphotericin B [96] shows this distinctness of antibiotic/antifungal MD simulations with CDs. First, in contrast to the majority of other cases, for such molecules, γ-CD (and not β-CD) is preferred. This is due to the size of the CD cavity space, which, in the case of β-CD, is not large enough; moreover, large guests simply cannot fit in it due to the steric hindrance. This fact was confirmed by the binding affinity calculations between amphotericin B and two CDs. Moreover, MD simulations and free energy calculations indicated that only the polyene macrolide ring could be included in CD. Second, due to the narrow and elongated macrolide ring of amphotericin B, both edges of the γ-CD (small and big) were observed as “available” for the drug to enter the host. Third, for each of these entering orientations, two possible binding sites were found. This is due to the fact that the macrolide ring is composed of repetitive structural elements and, therefore, many similar binding poses occur. Even if two binding sites differ in binding affinity, all of the complexes were reported as stable. The two mentioned binding sites are located on the amphotericin B at the distance of 12 Å, which is far enough to form a complex of two CDs attached to one amphotericin B at the same time (similar to two rings located on one pole). Interestingly, the binding site located more externally has higher binding affinity than the deeper one, located closer to the β-glycosidic moiety. Nevertheless, based on the MD simulation results, the authors suggest that, in high concentrations of an API, it could be possible to obtain a 2:1 host–guest complex of amphotericin B and γ-CD.

Similar results concerning the entering pose have been obtained for natamycin [92]. Namely, the best binding affinity shows the head-to-tail complex, which means that the CD is entered from the bigger edge (head) site by the elongated (tail) part of the guest. In this case, only native β-CD and its derivatives were taken into account, as the natamycin ring is smaller than the one of the amphotericin B.

A thorough analysis of a whole set of different CDs (α-CD, β-CD, γ-CD, and 2-HP-β-CD) was performed with cefuroxime axetil as a guest [93]. Here, the binding affinity was established as follows: 2-HP-β-CD ~ γ-CD > β-CD ~ α-CD. The explanation for this order was delivered by MD simulations. Both α-CD and β-CD interact with the furanyl ring of the drug, but 2-HP-β-CD and γ-CD are “outstanding” due to other bindings not present in α-CD- and β-CD-cefuroxime axetil complexes. Those interactions are enhanced by H-bonds in the case of 2-HP-β-CD (due to its hydroxypropyl groups) and interaction of the lactam ring with the CD, in case of γ-CD (due to the bigger γ-CD cavity when compared to other CDs), which stabilizes the complexes.

Another approach involves the application of MD simulation to foresee whether the CD-antibiotic complex could bind to the lipid membrane of potential bacteria. However, first, the CD-drug complex must be modeled. This was performed for γ-CD and alamethicin [94]. The structure is a peptide antibiotic with its *N*-terminal more hydrophobic than the *C*-terminal, which strongly influences the binding pose to the CD. This was confirmed during MD simulations.

### 3.5. Plant-Derived Substances

A practical use of encapsulation of plant-derived substances in CD complexes is either to enhance their solubility, as numerous plant substances include in their structures multiple aromatic rings, which distinctively lowers their solubility in water and, therefore, their bioavailability. This approach is also undertaken to enhance substance stability, more precisely: to protect plant-derived substances from the oxidative environment, because a lot of them should exert antioxidative effects in a human body. Thus far, approximately 30 articles taking into account plant-derivative-CD complexes with a MD approach have been published (Table 2: I D.). The reasons to apply this particular computational method include finding a preferable binding mode, establishing specific host–guest interactions and ways of entering the CD cavity, and obtaining ranked relative stability of various CD complexes 

**Figure 4:**
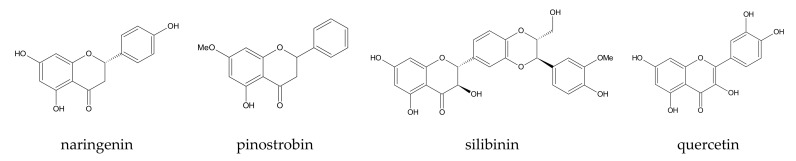
Examples of plant-derived compounds simulated in CD complexes with application of MD.

Often, there is similarity in the binding modes due to the structural closeness among the compounds, and such knowledge could be useful for choosing a proper CD for one’s calculations, which is one of the objectives of this review. Good examples include naringenin [65], pinostrobin [105], silibinin [121], and quercetin [120] (Figure 4). For both naringenin and pinostrobin, it was found that, in complexes with β-CD, only the chromone ring is included in the CD cavity, whereas when β-CD-derivatives are used, both chromone and phenyl ring hide in the CD cavity. Higher stability of the former complexes is supported by MMGBSA calculations.

Silibinin and quercetin exhibit different binding modes. Their condensed aromatic rings bond with the 2-HP-β-CD cavity via vdW interactions and the other aromatic rings stay outside stabilized via H-bonds made with the hydroxypropyl substituents from CD. For silibinin, this binding mode is easily explained by the fact that it is a bigger molecule. Quercetin resembles more naringenin and pinostrobin; however, its two hydroxyl groups attached to the aromatic non-condensed ring, making this part of a molecule too polar to enter the CD cavity. Even if H-bonds are formed outside the CD, they do not compensate vdW interactions, which are main binding forces in these complexes.

All of these results from MD simulations are in agreement with the NMR measurement results.

Among the wide range of simulations of CD complexes with plant-derivatives, there are examples that take into account the entropic effect. In most of the cases, it is calculated directly from the MD trajectories, as described in [109]. Less often, the calculations are performed on the snapshots from MD with QM methods, such as semi-empirical PM3 or even DFT [98].

In most of the cases, the configurational (and not thermal) entropy is calculated. It has a varied significance. For instance, in the glycyrrhizic acid-CD complex, configurational entropy was calculated for the bound and unbound state of β- and γ-CD complexes, and almost no differences between two complexes were observed upon the system’s alteration via complexation [102]. Therefore, in this system, an entropic component plays no role in the ranking of free energy binding of analyzed CDs. On the other hand, large entropy terms were obtained for α-mangostin complexed with β-, 2,6-DM-β-, and 2-HP-β-CD, which significantly influenced the stability order of those CD complexes [124,125].

One interesting entropy-inclusion study is the case of cannabidiol [109], where a temperature increase caused conformational changes in the simulated system and the cannabidiol got out of the CD cavity. The explanation is that the non-bonded interactions (vdW) are not sufficient to counterbalance the guest’s increasing temperature-dependent configurational entropy, which has a negative influence on stability. However, interestingly, in this system, entropy contribution is only relevant for α-CD and β-CD complexes, whereas γ-CD-cannabidiol is almost temperature-insensitive.

### 3.6. Others

In the ‘Others’ category (Table 2: I.E.), among guests, there are substances spanned from drugs to dyes [129,130,131]. Two works are worth mentioning, where CD with a guest (efavirenz or omeprazole) and L-arginine are simulated [127,128]. L-Arginine is positioned on the outer surface of CD and its role is to enhance the system’s solubility by increasing the complex’s polar sphere. What was discovered during the MD simulation is that L-arginine also increases the system stability. This occurs because L-arginine makes a bridge between the host and guest via H-bonds and electrostatic interactions. This study [127] is a very rare example among CD complexes using the TIP4P water model. TIP4P is characterized by the ability to improve the electrostatic distribution around water molecules [183]. However, it is also more computationally demanding.

Among all of the examples, we should point out the norepinephrine-CD study [147], as it clarifies the difference between the role of H-bonding and hydrophobic forces in, statistically, most cases of CD complexes. Namely, the former mainly ensures stability of the already created complex, whereas the latter, enables occurring of the inclusion process.

### 3.7. Umbrella Sampling and SMD Used for CD Simulations

Computational details regarding the works using SMD and/or umbrella sampling in the CD–guest systems are presented in Table 2: I.F. They were separated from other articles regardless of the guest type. There are two reasons for that. First, this calculation approach is plainly different when compared to a classical MD used in the other cases. Second, most of these works (7 out of 10) were published very recently, between 2018 and 2021, so they point out a new direction of CD–guest MD computation. Moreover, the complexity of this approach requires higher computational power in order to make the calculations reasonably long (or even simply feasible) to perform. Hence, they go hand-in-hand with the ongoing development of hardware.

One good example to start with is work by You et al. [69]. Regarding β-CD, 1-butanol and aspirin, they found that the starting host configuration could distinctively change the height of the energy barrier, which is seen as a change of the PMF depth change obtained from US. This cannot be corrected, even by a long (and biased) MD. This is why, in this study, more steps were implemented. First, in contrast to other works, an enhanced method sampling (other than US) was applied rather than a simple docking [63,167]. Second, a classical MD was performed. Third, the resulting trajectories were used for US in order to compute free energy along the CD–guest dissociation pathway. Another important conclusion was that, even if PMF values could distinctively change, depending on the initial CD conformation, the PMF pattern illustrating local minima and energy barriers always stays the same.

A similar approach was described in far earlier work, from 2016 [66]. There, first, a classical MD simulation was performed to establish how a guest enters the CD. Later, US was implemented to obtain more information on the inclusion complex formation and more detailed free energy of binding. Afterwards, on the geometries equilibrated during US, the ONIOM calculations were executed in order to obtain even more accurate energy values.

Referring to You’s paper [69] (the first article described in Section), another conclusion regarding the sensibility of initial assumptions and interpretation of SMD results was derived from the pinostrobin study. Initially classical MD simulations were conducted (see article from 2016 [105]) and later, inclusion complexes verified by this first attempt were applied to SMD simulations (see article from 2018 [166]). It was proven once more that one binding mode for β-CD (via chromone-ring) and two for β-CD derivatives (via phenyl- and chromone-ring) were obtainable (see the pinostrobin structure in Figure 4). The hypothesis was made that a higher pulling force corresponds to a more favorable guest orientation in the host cavity. The results show that, indeed, the differences in the pulling force are caused by the guest molecule orientations in the CD cavity.

We should reference the examples that use more complex three-element systems, such as adamantane-doxorubicin-CD [163] and adamantane-gold-CD [164], where SMD was applied, among others, to see if any conformational changes of the main component (doxorubicin and adamantane, respectively) occurred by creating a pro-drug (re: first case) or stabilizing the complex (re: second case). These conjugates were later simulated with CDs to observe the system properties as a solubility-enhancing drug carrier or oxidation-protecting agent.

Few articles published on CD–MD simulations using SMD/umbrella sampling could be quite rationally explained by the fact that these calculations are more complex, require much more time, computational power, and the researcher must be well-acquainted with these methods. 

### 3.8. CDs Used as Extracting Agents (Different Solvents)

Extraction is the second area in which CDs find application. In contrast to application of CDs as drug carriers, those processes lie much more in the industry sector, and sometimes solvents (other than water) must be used. This is why diversified solution environments are applied for the MD–CD simulations (Table 2 II).

The first topic involves separation of drug enantiomers. Here, two approaches are available. Either the classic method of simulating one guest with one CD, and checking for any selectivity among the tested CDs, as in the case of terbutaline enantiomers [178]. Or, in a more complicated approach, a channel or walls are formed out of multiple CDs; in this way, a kind of chromatographic column is simulated. An example case involves an ibuprofen racemic mixture [76]. It was shown that β-CD has different affinity towards *R*- and *S*-ibuprofen.

The second topic is extraction of pollutants from the environment. This includes both synthetic substances, such as polychlorinated biphenyls [170,171] and dioxins [169], as well as natural toxins, e.g., ochratoxin A [172]. A good example is a thorough MD study of DDT [171]. Both CD–DDT and CD–DDT-lipid membrane systems were simulated. For the latter, SMD was applied in order to force the DDT-dissolution from the CD–DDT complex on the membrane surface. An external force pulled DDT out of the CD–DDT complex. This way, the free energy landscape was calculated as a coordinate of the separation reaction. In this study, it was proven that DDT bound with all native CDs, and as a result, all CDs enhanced the DDT solubility in aqueous solvents. However, these bindings are characterized by different binding modes and affinities, with α-DDT being the least-strongly bonded complex.

The third topic is composed of other applications of CDs, for instance CDs as capping agents for gold nanoparticles (AuN) [177] or recognizing agents for *N*-methylfulleropyrrolidine regioisomers [179]. In those cases, multiple CDs are applied, regarding their roles. In the mentioned fullerene-derivatives study, one or two γ-CD molecules were used to separate the isomers. Whereas for AuN, γ-CD served as stabilizing agents; as an example: 1007 AuN and 60 CDs were simulated at once, in a way that they completely surrounded the guest.

## 4. Conclusions

As shown above, the number of works presenting the results of MD simulations on CD host–guest complexes have rapidly increased since the early 2010s. Moreover, the applied methods are becoming more sophisticated at increasing the accuracy of such calculation, to extend their application.

For example, while in the oldest works on this topic, the standard FFs were applied, currently, CD-dedicated FFs, such as Glycam06, are commonly used. Further, in the literature, examples of hybrid QM/MM methods, widely applied in protein–ligand interaction models, could be found. Similarly, solvent treatment methods are being improved. Initially, the implicit methods were used; however, they were found inaccurate and, thus, were replaced by explicit methods, such as TIP3P or even TIP4P.

MD of CD complexes are no longer simulated solely to analyze the RMSD or RMSF, but also for post-MD calculations, to better assess the binding and, thus, thermodynamic stability. Hence, GBSA or PBSA methods are commonly used. Moreover, very recent applications of umbrella sampling and steered (biased) MDs prove that state-of-the-art methods of MD could be useful in studying and designing CD complexes.

The versatility of MD simulations allows for studying all kinds of complexes. Both native and substituted CDs (as hosts) and a whole range of APIs (as guests) are being frequently modeled this way, in 1:1 and 2:1 molar ratios. Moreover, the application of CDs as extracting agents could also be evaluated by the means of MD simulations.

As shown in this review, MD simulations could be used to predict the structure, solubility, and stability of CD complexes and, thus, used to explain, at the molecular level, the experimental results. In addition, such simulations are now being used at the stage of the design of CD complexes, preceding their experimental preparations. With the anticipated progress in MD simulations, this second application is expected to become even more popular.

## Figures and Tables

**Table 1:** The most used software and force fields for MD simulation of CD complexes.

N°	Software/Code	Force Field	License	Ref.
1	GROMACS	GROMOS CHARMM Carbohydrate Solution force field, CSFF (for CD)	General Public License (GPL)	[32]
2	AMBER	GAFF, Glycam06 (for CD), q4md-CD (for CD)	Commercial	[33]
3	CHARMM	CHARMM FF	Commercial	[34]
4	Desmond (Schrödinger, Inc.)	OPLS	Commercial	[35]
5	NAMD	CHARMM	Commercial, academic	[36]
6	Forcite (BIOVIA Materials Studio, Accelrys)	COMPASS	Commercial	[37]
